# Mechanistic insights into dental stem cells‐derived exosomes in regenerative endodontics

**DOI:** 10.1111/iej.14269

**Published:** 2025-06-11

**Authors:** Paras Ahmad, Nathan Estrin, Nima Farshidfar, Yufeng Zhang, Richard J. Miron

**Affiliations:** ^1^ Department of Research Advanced PRF Education Jupiter Florida USA; ^2^ Department of Oral Biology, Rutgers School of Dental Medicine The State University of New Jersey Newark New Jersey USA; ^3^ Lake Erie College of Osteopathic Medicine School of Dental Medicine Bradenton Florida USA; ^4^ Department of Periodontology University of Bern Bern Switzerland; ^5^ Department of Oral Implantology University of Wuhan Wuhan China

**Keywords:** dental pulp, exosomes, regeneration, regenerative endodontics, stem cells

## Abstract

**Background:**

Dental pulp is a richly vascularised and innervated tissue vital for tooth vitality, sensory function, and structural integrity. While conventional root canal therapy effectively treats necrotic permanent teeth, it irreversibly eliminates pulp vitality, potentially increasing the risk of secondary infections and long‐term structural compromise. In response, regenerative endodontics has emerged as a biologically favourable alternative that seeks to restore the pulp–dentine complex using principles of tissue engineering.

**Objectives:**

This review aims to explore the therapeutic potential and mechanisms of action of exosomes derived from dental stem cells (DSC‐Exos), a subclass of mesenchymal stem cells (MSCs), in promoting regeneration of the pulp–dentine complex, while also addressing translational challenges and proposing an integrated regenerative framework.

**Methods:**

A comprehensive literature search was conducted across Web of Science, PubMed, and Scopus databases using keywords associated with “stem cells,” “exosomes,” “extracellular vesicles,” and “dental pulp regeneration.” Titles and abstracts were screened, and eligible studies were selected based on predefined inclusion criteria: (a) original research or case reports focusing on DSC‐Exos in regenerative endodontics, (b) in vitro and in vivo studies, and (c) clinical trials or animal studies showing pulp‐like tissue development. Studies not fulfilling these criteria were excluded. A total of 67 articles were included for narrative synthesis.

**Results:**

DSC‐Exos were found to facilitate multiple regenerative functions: promoting odontoblastic differentiation and dentine mineralisation, enhancing angiogenesis, regulating inflammation, modulating immune responses, promoting cell proliferation and migration, reducing apoptosis and senescence, and supporting neuroprotection. In‐vivo studies demonstrated pulp‐like tissue formation, revascularisation, and functional restoration. However, heterogeneity in exosome isolation, culture conditions, donor variability, and unclear molecular pathways remain unresolved issues.

**Discussion:**

DSC‐Exos present a promising acellular, immunologically safer approach to regenerative endodontics compared to direct stem cell transplantation. Despite their potential, the lack of standardised methodologies and incomplete understanding of their molecular interaction with odontoblasts hinders clinical translation. Integration of exosomes with scaffolds, growth factors, and endogenous cues may enhance regenerative efficacy.

**Conclusions:**

DSC‐Exos represent a novel frontier in regenerative endodontics. This review proposes a triangular framework encompassing DSCs, exosomes, signalling molecules, scaffolds, and the dentine microenvironment to support a holistic and clinically translatable model for pulp–dentine complex regeneration.

## INTRODUCTION

Dental pulp represents a tissue characterized by high innervation and vascularization that plays multiple roles, including responding to bacterial invasion and injury, and facilitating the neuronal sensitivity essential for the transmission of mechanical stimuli related to repair and regeneration. Thus, the absence of this tissue leads to a decline in tooth vitality (Farges et al., 2015). Currently, root canal treatment (RCT) is regarded as the preferred clinical intervention for the management of necrotic permanent teeth (Sabeti et al., 2024). Over 20 million RCTs are conducted annually in the United States alone, with several million more RCTs being performed worldwide (León‐López et al., 2022). The filling material used during RCT is an inorganic compound recognized for its biological inertness. Consequently, this intervention culminates in the irreversible loss of tooth vitality and sensitivity (termed a dead tooth) (Li, Guo, et al., 2021). The absence of an immune response in an insensate tooth may result in undetected subsequent infections, thereby increasing the risk of secondary infections (Cortellini & Tonetti, 2001). Thus, the preservation of dental pulp functionality is imperative for maintaining tooth integrity and longevity (Yu & Abbott, 2007).

Regenerative endodontics has emerged as a viable alternative to conventional RCT, aiming to restore the biological function of the pulp–dentine complex rather than simply filling the root canal with inert materials (Wei et al., 2022). These procedures leverage the principles of tissue engineering by utilizing stem cells, scaffolds and bioactive molecules to facilitate tissue regeneration (Albuquerque et al., 2014). The advantages of regenerative endodontics include the potential for continued root development, reestablishment of vascularization and restoration of pulp vitality, which collectively enhance the long‐term survival and mechanical properties of the tooth (Simon & Smith, 2014). Additionally, REP has demonstrated feasibility in clinical practice, particularly for immature necrotic teeth, offering a more biologically favourable approach than traditional RCT, which is primarily suitable for mature permanent teeth (Almalki, 2024; Elheeny et al., 2024).

Amongst the key components of REP, mesenchymal stem cells/medicinal signalling cells (MSCs) play a central role in regenerative endodontics. These cells contribute to tissue repair through their differentiation potential and secretion of bioactive factors that regulate inflammation, cell proliferation and angiogenesis (Merimi et al., 2021). MSCs can differentiate into odontoblast‐like cells, which are responsible for dentine formation and promote the migration and proliferation of endogenous progenitor cells, further enhancing regeneration (Liang et al., 2014). However, recent evidence suggests that the regenerative effects of MSCs are primarily mediated by their secretome, which includes extracellular vesicles (EVs), particularly exosomes (Kou et al., 2022).

Exosomes are small EVs, approximately 30–150 nm in diameter, that originate from intracellular multivesicular bodies. They encapsulate bioactive molecules such as proteins, lipids, transcription factors and nucleic acids (including microRNAs [miRNAs], messenger RNAs and DNA), which contribute to intercellular communication and influence the behaviour of recipient cells (Ahmad et al., 2025; Miron & Zhang, 2024). Exosomes exert their biological effects by interacting with cell surface receptors or fusing with the membrane of target cells, modulating cellular activities such as apoptosis inhibition, inflammation reduction, proliferation enhancement and angiogenesis stimulation (Estrin et al., 2025; Miron et al., 2024a, 2024b; Monje et al., 2022).

In the context of regenerative endodontics, dental stem cell‐derived exosomes (DSC‐Exos) have emerged as a promising acellular therapeutic approach. These exosomes can modulate the local dental microenvironment, facilitating regenerative endodontics without the potential risks associated with direct stem cell transplantation, such as immune rejection or uncontrolled differentiation (Ning et al., 2024). Whilst significant advancements have been made in DSC‐Exo isolation techniques and scaffold development (Huang et al., 2021), the reconstruction of a functional pulp–dentine complex remains a challenge. Incomplete restoration of the microenvironment is a major limiting factor in regenerative outcomes, highlighting the need for strategies that enhance endogenous tissue responses (Mai et al., 2021).

Addressing this challenge might involve optimizing the biological properties of DSC‐Exos to increase their regenerative effects, leveraging bioactive molecules or stimulating endogenous stem cells to improve microenvironmental conditions. As a minimally invasive and biologically potent approach, DSC‐Exos hold significant potential in redefining regenerative endodontics. Thus, the present review explores the regenerative potential of DSC‐Exo‐based therapies in regenerative endodontics, focusing on their mechanisms of action and outcomes in in vitro and in vivo studies.

## METHODS

An electronic search of the literature was conducted with Clarivate Analytics' Web of Science (All Databases), PubMed via MEDLINE and Elsevier's Scopus databases to identify articles associated with regenerative endodontics without any limitations on the year or language of publication. The keywords utilized in the preliminary search included ‘stem cells’, ‘exosomes’, ‘extracellular vesicles’ and ‘dental pulp regeneration’. The collected literature was initially evaluated on the basis of the title and abstract, followed by a thorough examination of the content. This review offers an extensive and detailed narrative review of the literature that met the final screening criteria. The inclusion parameters were as follows (Kong, Li, et al., 2024): (a) original research articles and case reports about regenerative endodontics with DSC‐Exos; (b) comprehensive clinical studies and investigations conducted on cellular or animal models (Tibúrcio‐Machado et al., 2021); (c) animal experimentation must exhibit the development of pulp‐like tissue in vivo (Xie et al., 2021); and (d) clinical trials related to dental pulp tissue engineering with DSC‐Exos comprising both randomized clinical trials and case reports (Kim et al., 2018). Publications unable to satisfy the inclusion criteria were excluded from this study.

## RESULTS

**FIGURE 1:**
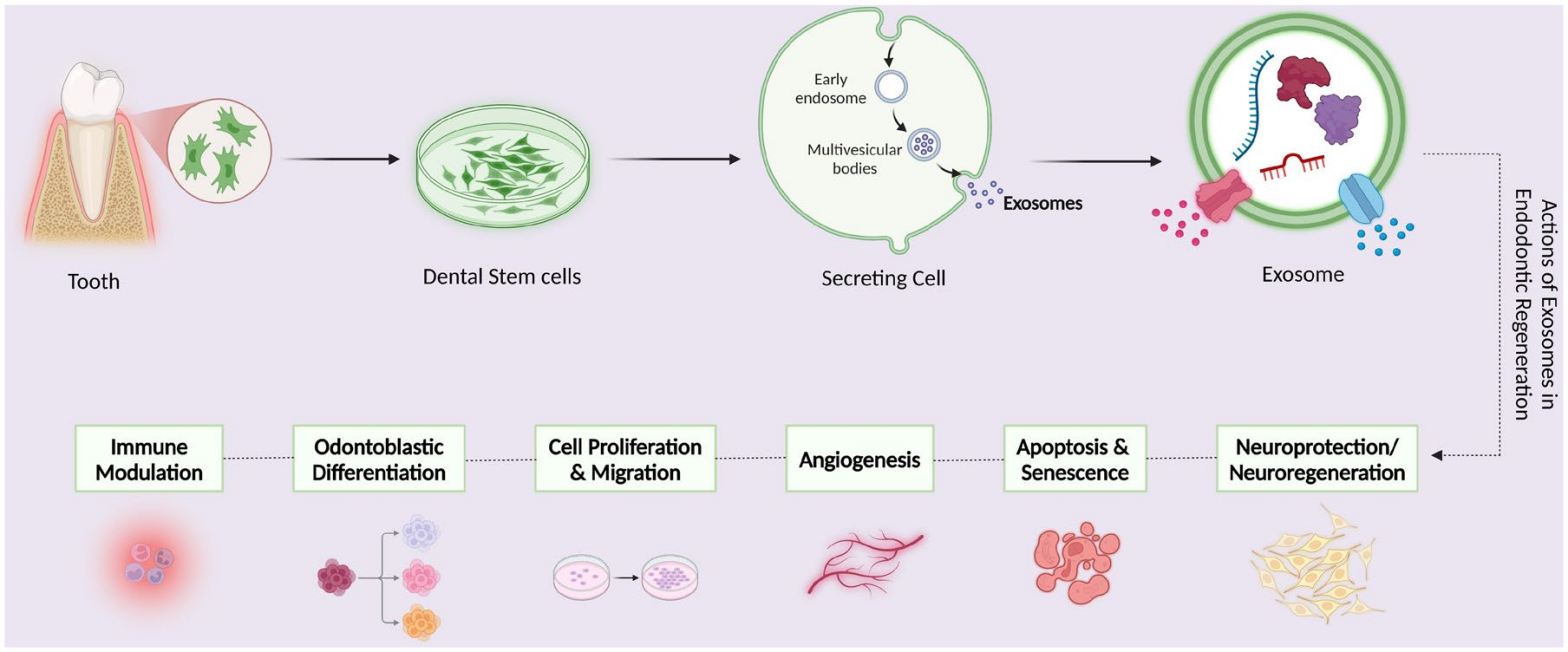
Exosomes are formed through a process involving the endosomal system in which proteins, nucleic acids or lipids accumulate at the endosomal membrane. This accumulation triggers the inward budding of the late‐sorting endosomal membrane, resulting in the formation of intraluminal vesicles. These exosomes are subsequently taken up by recipient cells via mechanisms such as endocytosis, receptor–ligand interactions and fusion. Once internalized, exosomes can influence various cellular processes in pulp healing and regeneration, including immunomodulation, odontoblastic differentiation and dentine formation, cell proliferation and migration, angiogenesis, apoptosis and senescence and neuroregeneration.

According to the findings of this review, DSC‐Exos contribute to regenerative endodontics via several biological processes, such as (1) promoting dentinogenesis; (2) increasing angiogenesis; (3) regulating inflammation, repair and immune responses; (4) mediating cell proliferation and migration; (5) modulating apoptosis and senescence; and (6) providing neuroprotection or neuroregeneration (Figure 1).

### Odontoblastic differentiation and dentine mineralization

To achieve comprehensive structural and functional regeneration of the pulp–dentine complex, it is essential to activate the multilineage differentiation capabilities of stem cells (Grottkau et al., 2010). When MSCs are either recruited to or implanted at the site of pulp injury, the primary objective is their differentiation into odontoblasts, which subsequently leads to the formation of tubular dentine, thereby facilitating the repair of compromised hard dental tissue (Pan et al., 2023). This process does not occur in isolation; it is intrinsically linked to angiogenesis, cell migration and immune regulation, which collectively establish a conducive microenvironment for regeneration. The interplay between these mechanisms ensures the continuous supply of nutrients, immune modulation to mitigate excessive inflammation and increased cellular recruitment, all of which are essential for effective pulp–dentine complex restoration (Xie et al., 2021). Consequently, the induction of odontogenic differentiation in MSCs has emerged as a pivotal focus for regenerative endodontics (Huang et al., 2021).

Numerous studies have indicated that exosomes can increase the multidirectional differentiation of stem cells, thus providing them with considerable potential for regenerative endodontics (Abdik et al., 2019). For example, angiogenic factors secreted by exosome‐treated endothelial cells not only improve vascularization but also act as signalling molecules that increase odontoblastic differentiation, highlighting the bidirectional relationship between angiogenesis and dentine mineralization (Zhuang et al., 2020). Similarly, exosomes encapsulating nuclear factor I/C were engineered to reinstate odontoblastic differentiation whilst concurrently increasing the proliferation and migration of stem cells from the apical papilla (SCAPs), emphasizing the dynamic interaction between cell migration and odontogenesis (Yang et al., 2022).

Moreover, exosomes preconditioned from stem cells from human exfoliated deciduous teeth (SHEDs) exhibited increased therapeutic efficacy, particularly when incorporated into photocrosslinkable hydrogels, which ensured sustained release, improved biocompatibility and increased odontogenic differentiation of dental pulp stem cells (DPSCs) (Lu et al., 2024). This finding demonstrates how biomaterial‐based strategies can be leveraged to optimize exosome‐mediated regenerative outcomes, bridging the gap between cellular differentiation and scaffold‐supported regeneration. In another study, exosomes derived from dental pulp cells under odontogenic stimuli effectively initiated lineage‐specific differentiation of DPSCs and bone marrow mesenchymal stem cells (BMMSCs) through the p38 mitogen‐activated protein kinase (MAPK) signalling pathway, culminating in significant regeneration of pulp‐like tissue (Huang et al., 2016).

Advancements in delivery approaches, such as the utilization of an amphiphilic triblock copolymer for the regulated release of exosomes, have been shown to further promote dentine bridge formation, surpassing the efficacy of conventional materials in vivo (Swanson et al., 2020). Interestingly, similar controlled‐release strategies have been applied in angiogenesis‐focused therapies, underscoring the potential of integrated approaches that simultaneously increase vascularization and odontogenesis (Divband et al., 2022; Ruan et al., 2023). Additionally, the integration of photobiomodulation with DPSC‐Exos synergistically increased the mineral content and upregulated critical regenerative markers in a canine model (Abdelgawad et al., 2022), demonstrating convergence between exosome‐mediated differentiation and light‐induced cellular responses.

Diomede et al. (2022) reported the efficacy of a decellularized dental pulp (DPP) matrix that was cotreated with exosomes and 5‐aza‐2'‐deoxycytidine which significantly improved dentinogenic differentiation, underscoring the potential applicability of DPP‐based scaffolds in regenerative endodontics. Similarly, a thermosensitive hydrogel composed of hydroxypropyl chitin/chitin whiskers and infused with exosomes has emerged as a promising substitute for traditional RCT, exhibiting strong in vitro and in vivo outcomes in facilitating odontogenesis and the formation of pulp‐like tissues (Wang et al., 2023). Another study highlighted the influence of exosomes derived from lipopolysaccharide (LPS)‐preconditioned DPSCs on improving Schwann cell migration, proliferation and odontogenic differentiation, thereby facilitating dentin repair through intercellular communication (Li, Ju, et al., 2021).

Exosomes generated under odontogenic conditions were observed to increase differentiation via the transforming growth factor beta 1 (TGF‐β1)/suppressor of mothers against decapentaplegic (SMAD) signalling pathway, with miRNAs such as miR‐27a‐5p downregulating inhibitory factors, including latent transforming growth factor beta binding protein 1 (LTBP1) (Hu et al., 2019). Moreover, the mechanistic target of rapamycin complex 1 (mTORC1) was demonstrated to govern the release of exosomes from odontoblasts, influencing their inhibitory effect on DPSC differentiation and providing valuable insights into the equilibrium of the cellular signalling necessary for effective regeneration (Luo et al., 2023). This highlights a regulatory network in which signalling pathways do not act in isolation but rather coordinate with exosomal activity to modulate the balance between differentiation and mineralization.

Furthermore, miR‐148a‐3p is recognized as a fundamental regulator of odontoblastic differentiation, modulating the wingless‐related integration site 1 (Wnt1)/β‐catenin pathway and affecting DPSC viability and invasive capacity (Li & Huang, 2021). Moreover, miR‐223‐3p is associated with pulpitis repair, and its upregulation facilitates odontoblastic differentiation by inhibiting SMAD3, thus promoting increased expression of the dentine‐specific proteins dentine sialophosphoprotein (DSPP) and dentine matrix acidic phosphoprotein 1 (DMP‐1) (Huang et al., 2019). These findings reinforce the link between immune regulation and odontogenesis, as inflammation‐responsive miRNAs play crucial roles in dictating differentiation outcomes.

The long noncoding RNA (lncRNA) calbindin 2 (CALB2) has been shown to facilitate odontoblastic differentiation by sequestering miR‐30b‐3p, which subsequently leads to the upregulation of runt‐related transcription factor 2 (RUNX2) expression (Tu et al., 2020); conversely, miR‐143‐5p acts as an inhibitor of differentiation by downregulating the p38 MAPK pathway, and its reduced expression correlates with increased odontogenic markers and mineralization (Wang, Wang, et al., 2019). The lncRNA insulin‐like growth factor binding protein 7 antisense RNA 1 (IGFBP7‐AS1) facilitates SHED odontogenic differentiation by decreasing the repressive effects of miR‐335‐3p and miR‐155‐5p on the extracellular signal‐regulated kinase (ERK) pathway (Zhu et al., 2023). Interestingly, the ERK pathway is also pivotal in angiogenesis and cell migration, reinforcing the concept that these biological mechanisms operate in concert (Samson et al., 2022; Song et al., 2023).

Additionally, miR‐140‐5p exerts a negative effect on odontoblastic differentiation through targeting of the Wnt1/β‐catenin pathway, and its inhibition can counteract this suppressive effect (Lu et al., 2019). The lncRNA small nucleolar RNA host gene 1 (SNHG1) has been demonstrated to promote odontogenic differentiation by sponging miR‐328‐3p, thus activating the Wnt1/β‐catenin pathway (Fu et al., 2022), whereas the miR‐21/signal transducer and activator of transcription 3 (STAT3) signalling axis increases differentiation under conditions of low‐dose tumour necrosis factor alpha (TNF‐α), with miR‐21 serving as a regulatory factor (Xu et al., 2018). Notably, STAT3 signalling also plays a role in immune responses, further emphasizing the interconnected nature of differentiation, inflammation and tissue repair (Hillmer et al., 2016).

Moreover, the lncRNA lymphoid enhancer‐binding factor 1 antisense RNA 1 (LEF1‐AS1) augmented osteogenic processes in DPSCs by sequestering miR‐24‐3p, leading to the upregulation of transforming growth factor beta receptor 1 (TGF‐βR1) expression (Wu et al., 2020) (Table 1). Taken together, these studies reveal a highly integrated biological landscape in which odontoblastic differentiation, angiogenesis, cellular migration and immune modulation form an interdependent network, ultimately ensuring successful regenerative endodontics.

**TABLE 1:** Summary of studies assessing the role of exosomes derived from dental stem cells in odontoblastic differentiation and dentin mineralization.

Study	Objective	Exosome/miRNA source	Pathway	Application	Key findings
Zhuang et al. (2020)	To explore SCAP‐Exos in dentine–pulp complex regeneration	SCAP‐Exos	SCAP‐Exo→improved dentinogenesis of BMMSCs in vitro and in vivo	Potential therapeutic application in regenerative endodontic procedures for dentine–pulp complex formation	SCAP‐Exos enhances dentinogenesis in BMMSCs by increasing gene/protein expression of DSPP and mineralized nodule formation
Yang et al. (2022)	To assess Exos in apical periodontitis and their role in SCAP odontoblastic differentiation	Exos with encapsulated NFIC	NFIC‐encapsulated Exos→increased NFIC levels→improved SCAP proliferation/migration and dentinogenesis	Exos as delivery vehicles for promoting dentin regeneration in apical periodontitis	Exos from inflamed pulp suppress odontoblastic differentiation of SCAPs, but NFIC‐encapsulated Exos promotes dentinogenesis
Lu et al. (2024)	To evaluate preconditioned SHED‐Exos (OM‐Exos) combined with a hydrogel in pulp regeneration	SHED‐Exos preconditioned with an odontogenic induction medium	OM‐Exos→AMPK/mTOR pathway activation→enhanced odontogenesis	Injectable OM‐Exos‐hydrogel system for improved pulp regeneration and dentin repair	OM‐Exos promotes odontogenic differentiation and dentinogenesis of DPSCs via the AMPK/mTOR pathway
Huang et al. (2016)	To assess Exos as biomimetic tools for odontogenic differentiation of stem cells	Exos from DPSCs cultured under odontogenic conditions	Odontogenic Exos→p38 MAPK pathway→enhanced odontogenic gene expression and mineralization	Exos as effective tools for dental pulp‐like tissue regeneration	Exos derived under odontogenic conditions induce lineage‐specific differentiation via the p38 MAPK pathway
Swanson et al. (2020)	To develop a controlled‐release platform for odontogenic exosomes in dentin bridge formation	Exos from DPSCs and MDPC‐23 cells	Exos‐loaded polymer→sustained release→improved odontogenic response	Controlled delivery system for enhanced dentin bridge formation in regenerative endodontics	Exos encapsulated in a triblock copolymer facilitate reparative dentin bridge formation superior to conventional materials
Abdelgawad et al. (2022)	To evaluate PBM and Exos for pulp regeneration in dogs	DPSC‐Exos	PBM + Exos→improved biochemical and genetic markers of pulp regeneration	Promising approach to augment pulp regeneration using PBM and Exos in dental therapies	PBM combined with Exos enhances mineral content (Ca, P, ALP) and increases gene expression (MMP9, TGF‐β, OCN) during pulp regeneration
Diomede et al. (2022)	To develop a growth‐permissive system for dental pulp regeneration using DDP matrix, 5‐Aza and DPSCs‐Exos	DPSC‐Exos in combination with 5‐Aza	Exos+5‐Aza→enhanced hDPSC differentiation on DDP→upregulated dentinogenic protein expression	A potential therapeutic system for dental pulp regeneration and endodontic applications	Co‐treatment with Exos and 5‐Aza significantly enhanced dentinogenic protein expression (ALP, RUNX2, COL1A1, DMP1, DSPP)
Wang et al. (2023)	To evaluate an Exos‐loaded hydrogel as a scaffold for dental pulp regeneration in devitalized human teeth	Exos from DPSCs embedded in thermosensitive HPCH/CW hydrogel	Exos‐loaded hydrogel→sustained delivery→improved odontogenic and angiogenic responses	Injectable hydrogel system as an alternative to traditional root canal therapy in dental clinics	HPCH/CW/Exos hydrogel enhances odontogenesis, angiogenesis and dental pulp‐like tissue formation in vivo
Li, Ju, et al. (2021)	To investigate the effects of DPSC‐Exos on SC proliferation, migration and odontogenic differentiation	Exos from LPS‐preconditioned hDPSCs	LPS‐Exos→improved SC migration and dentinogenesis	Potential role in modulating dental repair mechanisms via Exos‐based therapies	LPS‐preconditioned DPSC‐Exos (LPS‐exo) had enhanced effects on SC migration and differentiation compared to regular Exos
Hu et al. (2019)	To explore the regulatory mechanisms of miRNAs in Exos from DPSCs under odontogenic conditions	Exos from DPSCs under odontogenic conditions enriched with miR‐27a‐5p	OD‐Exos→miR‐27a‐5p→TGFβ1/Smads signalling→enhanced odontogenesis	Insight into miRNA‐mediated signalling cascades for promoting dental tissue regeneration	OD‐Exos containing miR‐27a‐5p activates TGFβ1/Smads signalling, promoting odontogenic differentiation by downregulating LTBP1
Luo et al. (2023)	To understand the role of mTORC1 in regulating Exos release from odontoblasts and their effect on DPSC differentiation	Exos from MDPC23 cells with variable mTORC1 activity	mTORC1 activity→modulation of Exos release without altering content→inhibition of DPSC differentiation	New insights into mTORC1‐mediated Exos regulation and its role in dental pulp regeneration	mTORC1 regulates Exos release from odontoblasts, and exosomes inhibit DPSC differentiation in a concentration‐dependent manner

Study	miRNA involved	Key genes involved	Regulatory mechanism	Significance	Key findings
Li and Huang (2021)	miR‐148a‐3p	Wnt1, β‐catenin, DSPP, DMP‐1, RUNX2, OCN, SMAD4	miR‐148a‐3p suppresses the Wnt1/β‐catenin pathway, reducing odontoblast differentiation markers, whilst its inhibition or Wnt1 overexpression enhances differentiation	miR‐148a‐3p/Wnt1/β‐catenin pathway is critical for regulating odontoblast differentiation and may guide therapeutic interventions	miR‐148a‐3p inhibits odontoblast differentiation of DPSCs, reducing markers such as DSPP and DMP‐1 Wnt1 is a direct target of miR‐148a‐3p
Huang et al. (2019)	miR‐223‐3p	SMAD3, DSPP, DMP‐1, ALP	miR‐223‐3p targets SMAD3, reducing its expression, which enhances odontoblast differentiation and promotes pulpitis repair	miR‐223‐3p/SMAD3 axis is a potential therapeutic target for pulpitis and odontoblast regeneration	miR‐223‐3p promotes odontoblast differentiation in inflamed dental pulp tissues. Overexpression increases DSPP, DMP‐1 and ALP whilst reducing SMAD3 protein levels
Tu et al. (2020)	miR‐30b‐3p	CALB2, RUNX2	CALB2 acts as a sponge for miR‐30b‐3p, upregulating RUNX2, which promotes differentiation	CALB2/miR‐30b‐3p/RUNX2 axis could be a therapeutic target for odontogenesis	CALB2 promotes odontoblast differentiation in DPSCs
Wang, Wang, et al. (2019)	miR‐143‐5p	MAPK14, Ras, MKK3/6, ALP, DSPP, OCN	miR‐143‐5p negatively regulates MAPK14 and odontoblast differentiation markers	miR‐143‐5p could be targeted to enhance odontogenesis via p38 MAPK pathway	miR‐143‐5p regulates odontoblast differentiation via p38 MAPK pathway
Zhu et al. (2023)	miR‐335‐3p, miR‐155‐5p	IGFBP7‐AS1, DSPP	miR‐335‐3p and miR‐155‐5p inhibit IGFBP7‐AS1‐mediated differentiation via ERK pathway suppression	miR‐335‐3p and miR‐155‐5p are negative regulators in SHED differentiation	IGFBP7‐AS1 promotes SHED odontogenesis through miR‐335‐3p and miR‐155‐5p
Lu et al. (2019)	miR‐140‐5p	Wnt1, β‐catenin	miR‐140‐5p targets Wnt1, inhibiting odontoblastic differentiation	miR‐140‐5p is a key regulator of odontogenesis via Wnt/β‐catenin pathway	miR‐140‐5p regulates odontoblastic differentiation via Wnt/β‐catenin signalling
Fu et al. (2022)	miR‐328‐3p	SNHG1, DSPP, DMP‐1, ALP	SNHG1 binds miR‐328‐3p, inhibiting Wnt/β‐catenin signalling, promoting differentiation	SNHG1/miR‐328‐3p/Wnt/β‐catenin pathway may be targeted for enhanced odontogenesis	SNHG1 promotes odontogenesis in hDPSCs via miR‐328‐3p and Wnt/β‐catenin pathway
Xu et al. (2018)	miR‐21	STAT3, miR‐21	miR‐21 interacts with STAT3 to modulate odontoblast differentiation, regulated by TNF‐α	miR‐21/STAT3 axis is a new target for odontogenesis modulation	miR‐21 regulates odontoblast differentiation of DPSCs through STAT3
Wu et al. (2020)	miR‐24‐3p	LEF1‐AS1, TGFBR1	LEF1‐AS1 acts as a sponge for miR‐24‐3p, regulating TGFBR1 for osteogenesis	LEF1‐AS1/miR‐24‐3p/TGFBR1 pathway could be targeted for bone regeneration in dental pulp	LEF1‐AS1 promotes osteogenic differentiation of DPSCs via miR‐24‐3p

### Neuroprotection/neuroregeneration

Numerous reviews have shown that MSC‐Exos might play a significant role in neurogenesis, neurogenic niches and therapeutic approaches for neurological disorders (Jarmalavičiūtė et al., 2015; Luarte et al., 2016; Yang et al., 2017). In regenerative endodontics, only one study (Zhang, Yang, et al., 2020) has been conducted to date, which reported that exosome‐like vesicles derived from a Hertwig's epithelial root sheath (HERS) cell line (ELVs‐H1) increased the tube‐like formation of human umbilical vein endothelial cells (HUVECs) and concurrently resulted in an increase in the expression of neurogenic markers in dental papilla cells (DPCs) in vitro. These findings suggest a dynamic interplay between angiogenesis and neurogenesis, where the vascular network not only supplies nutrients but also provides essential signalling cues for neurogenic differentiation.

ELVs‐H1 can, at least in part, replicate the functions of their progenitor cells in promoting the differentiation of mesenchymal cells and the synthesis of the extracellular matrix (ECM). Given the ability of DSC‐Exos to regulate neuroinflammatory pathways and increase neuronal survival in extradental applications, these exosomes may contribute to pulp nutritional function recovery by modulating sensory nerve regeneration, restoring neural connectivity and mitigating neuroinflammatory damage following pulp injury (Ge et al., 2024; Li, Sun, et al., 2022). Further studies are warranted to elucidate their potential role in functional nerve repair within the pulp–dentine complex. By acting as mediators in mesenchymal–epithelial interactions, these exosomes coordinate odontogenic, angiogenic and neurogenic differentiation, reinforcing the necessity of a well‐orchestrated microenvironment for effective regenerative endodontics.

In addition to regenerative endodontics, the ability of DSC‐Exos to modulate neuroinflammation (Liang et al., 2024), increase neuronal survival (Mai et al., 2021) and promote axonal regeneration (Chai et al., 2024; Li, Duan, et al., 2022) has been explored in various neurological contexts. For example, DPSC‐Exos exhibit neuroprotective effects in models of spinal cord injury and ischaemic stroke by suppressing oxidative stress and apoptosis whilst promoting neuronal differentiation (Jarmalavičiūtė et al., 2015; Zou et al., 2023).

### Apoptosis and senescence

Odontoblasts exhibit stability and are organized in a tightly packed arrangement within the human dental pulp (Kawashima & Okiji, 2016). The ability of odontoblasts to sustain their viability is crucial for the outcome of inflamed dental pulp during the advancement of dental caries (Farges et al., 2013). A research group led by L. Zhang (Pei et al., 2015, 2016; Wang et al., 2016) reported that autophagy serves as an intercellular mechanism through which odontoblasts treated with LPS engage in an anti‐apoptotic response. Notably, the inflammatory microenvironment induced by LPS is highly heterogeneous, influencing cellular responses in a location‐dependent manner within the pulp (El Karim et al., 2021; Galler et al., 2021; Rodas‐Junco et al., 2024). Certain odontoblasts situated in proximity to or directly adjacent to carious lesions exhibit markedly more pronounced alterations in response to LPS stimulation, whereas the most distant odontoblasts demonstrate comparatively milder changes.

As a cohesive protective barrier, preservation of the odontoblast layer is vital for its resilience against external stimuli. However, it remained unclear whether an intercellular mechanism existed that safeguarded the closely associated odontoblasts from such external influences until 2019, when the same research group confirmed that odontoblasts indeed secrete exosomes, particularly in inflammatory niches (Wang, Yang, et al., 2019). This group demonstrated that LPS treatment augmented exosome synthesis in human SCAP‐derived odontoblast‐like cells subjected to mineralization medium. The exosomes derived from LPS‐treated odontoblast‐like cells exhibited anti‐apoptotic properties (Wang, Yang, et al., 2019). These findings suggest that odontoblast‐derived exosomes may serve as protective agents, contributing to a self‐regulating network that mitigates inflammatory damage and promotes tissue homeostasis, linking immune responses to cellular survival mechanisms.

### Cell proliferation and migration

A critical first step in regenerative endodontics is the migration and proliferation of local MSCs to the damaged site (Kwack & Lee, 2022). From a clinical standpoint, the migration of cells towards the dentine interface is as critical as the inherent capabilities of the MSC. If the cells fail to access the dentinal wall, the establishment of a healthy pulp–dentine complex becomes unattainable (Shah et al., 2020; Wang, Huang, & Dong, 2020).

DSC‐Exos represent a promising cell‐free therapeutic modality for regenerative endodontics. Their capacity to recruit and augment the functionality of host cells, including DPSCs and SCAPs, has undergone extensive research. Compared with those derived from other sources, exosomes derived from dental pulp tissue (DPT‐Exos) exhibit superior efficacy in increasing the migration, proliferation and differentiation of SCAPs (Chen et al., 2022). These observations have been corroborated through both in vitro and in vivo studies in which DPT‐Exos facilitated the regeneration of dental pulp characterized by collagen, odontoblasts and vascularized structures (Chen et al., 2022).

Moreover, miR‐224‐5p (Ke et al., 2019) and miR‐140‐5p (Sun et al., 2017) have been recognized as significant modulators that impact DPSC migration, proliferation and differentiation. The downregulation of miR‐224‐5p and the upregulation of miR‐140‐5p significantly increased DPSC proliferation, whilst miR‐140‐5p also exerted a negative regulatory effect on differentiation by interacting with toll‐like receptor 4 (TLR‐4) (Ke et al., 2019; Sun et al., 2017). Similarly, DPSC‐Exos promoted the migration and proliferation of MSCs through lncRNA‐ankyrin repeat domain‐containing protein 26 (Ankrd26)‐mediated regulation of the miR‐150/TLR‐4 signalling pathway (Li & Ge, 2022).

The interaction between miRNAs and signalling pathways, including the protein kinase B (Akt) pathway, further elucidates the mechanisms that govern DPSC proliferation and migration (Tian et al., 2020). For example, miR‐584 was found to inhibit DPSC proliferation by directly targeting the transcriptional coactivator with PDZ‐binding motif (TAZ), a transcriptional coactivator essential for cellular growth, thereby suppressing cell cycle‐associated proteins. Altering miR‐584 levels or overexpressing TAZ was shown to restore proliferative ability, suggesting further therapeutic strategies for regenerative endodontics (Tian et al., 2020). These effects are mediated not only through exosome–cell interactions but also through the modulation of signalling pathways, including the Notch, Akt and TLR‐4 pathways, which govern cellular behaviour during regeneration (Ha et al., 2020; Mai et al., 2021; Tienda‐Vázquez et al., 2023).

The amalgamation of exosomes with fibrin gel delivery systems revealed increased efficacy in attracting MSCs and facilitating their proliferation (Ivica et al., 2020). Such biomaterial‐integrated approaches underscore the necessity of a combined strategy in which exosome signalling, scaffold‐mediated cell recruitment and angiogenic factors work synergistically to increase tissue repair. Furthermore, the differential expression of genes associated with the Notch signalling pathway between SCAPs and DPSCs underscores the importance of molecular signalling in modulating cellular behaviours, with SCAPs exhibiting increased proliferative and colony‐forming potential, whereas DPSCs presented increased expression of Notch‐related genes (Damrongsri et al., 2021) (Table 2).

**TABLE 2:** Summary of studies assessing the role of exosomes derived from dental stem cells in cell proliferation and migration.

Study	Objective	Exosome/miRNA source	Pathway	Application	Key findings
Chen et al. (2022)	To determine whether DPT‐Exos can regenerate dental pulp by recruiting SCAPs	Exosomes from dental pulp tissue and dental pulp stem cells	Recruitment of SCAPs by DPT‐Exos to the pulp cavity to regenerate connective and predentin‐like tissues	Demonstrated potential for treating pulp deficiency via cell homing for dental pulp regeneration	DPT‐Exos outperformed DPC‐Exos in promoting migration, proliferation and differentiation of SCAPs in vitro and in regenerating dental pulp‐like tissue in vivo
Ivica et al. (2020)	To explore the use of dental pulp‐derived Exos for cell‐free regenerative endodontics	Exosomes derived from third‐molar pulp cells	MSC migration and proliferation stimulated by dental pulp‐derived Exos	Highlighted the potential of Exos‐fibrin gel combination for safer and more effective regenerative endodontics	Pulp‐Exos attracted MSCs, enhanced their proliferation and demonstrated improved efficacy when combined with a fibrin gel

Study	miRNA involved	Key genes involved	Regulatory mechanism	Significance	Key findings
Li and Ge (2022)	miR‐150	lncRNA‐Ankrd26, TLR4	lncRNA‐Ankrd26 regulates miR‐150/TLR4 signalling to enhance migration and differentiation of MSCs	Identifies therapeutic targets for dental pulp repair and pulpitis treatment	DPSCs‐derived exosomal lncRNA‐Ankrd26 promotes dental pulp restoration by regulating MSC migration and osteoblastic differentiation
Ke et al. (2019)	miR‐224‐5p	SCAP, DPSCs	miR‐224‐5p negatively regulates DPSCs functions, suggesting its modulation as a strategy for pulp regeneration	Advances regenerative endodontics by promoting cell homing in pulp regeneration therapies	miR‐224‐5p downregulation enhances DPSCs proliferation and migration, offering an alternative to SCAP for regenerative endodontics
Sun et al. (2017)	miR‐140‐5p	TLR‐4	miR‐140‐5p binds to TLR‐4 mRNA 3′UTR, regulating DPSC proliferation and differentiation	Highlights miR‐140‐5p as a regulator in DPSC‐mediated tissue engineering	miR‐140‐5p inhibits DPSC differentiation whilst promoting proliferation by targeting TLR‐4
Tian et al. (2020)	miR‐584	TAZ	miR‐584 directly targets TAZ mRNA 3′UTR, regulating cell cycle proteins through Akt signalling pathway	Potential biomarker and therapeutic target for dental pulp diseases	miR‐584 inhibits DPSCs proliferation and migration by suppressing TAZ expression and Akt signalling
Damrongsri et al. (2021)	Not specified	NOTCH pathway genes	Differential expression of Notch‐related genes influences proliferation and colony formation of DPSCs and SCAPs	Enhances understanding of Notch signalling in dental pulp and apical papilla stem cells	Coronal pulp and apical papilla exhibit distinct Notch signalling profiles, affecting stemness and behaviours of DPSCs and SCAPs

### Angiogenesis

Angiogenesis plays a crucial role in the growth and repair of the pulp–dentine complex. In the context of regenerative endodontics, the limited vascular access within the tooth, restricted by the dimensions of the apical foramen, leads to inadequate revascularization, which hinders the regeneration process (Huang et al., 2013). Consequently, ensuring an adequate blood supply represents one of the primary challenges to address. Importantly, the process of vascularization is tightly linked to other regenerative mechanisms, as newly formed blood vessels not only transport essential nutrients but also deliver molecular signals that regulate cell proliferation, differentiation and immune responses (Shi et al., 2023; Xie et al., 2016).

Exosomes harvested from hypoxia‐preconditioned SCAPs promote angiogenesis in HUVECs via the hypoxia‐inducible factor 1 alpha (HIF‐1α)/Notch/vascular endothelial growth factor (VEGF) signalling pathway, thereby significantly increasing cellular proliferation, migration and tubular structure formation (Liu et al., 2023). In turn, angiogenesis creates a regenerative niche that facilitates odontoblast differentiation and dentine formation, reinforcing the interconnected nature of these processes (Matsubara et al., 2022).

**FIGURE 2:**
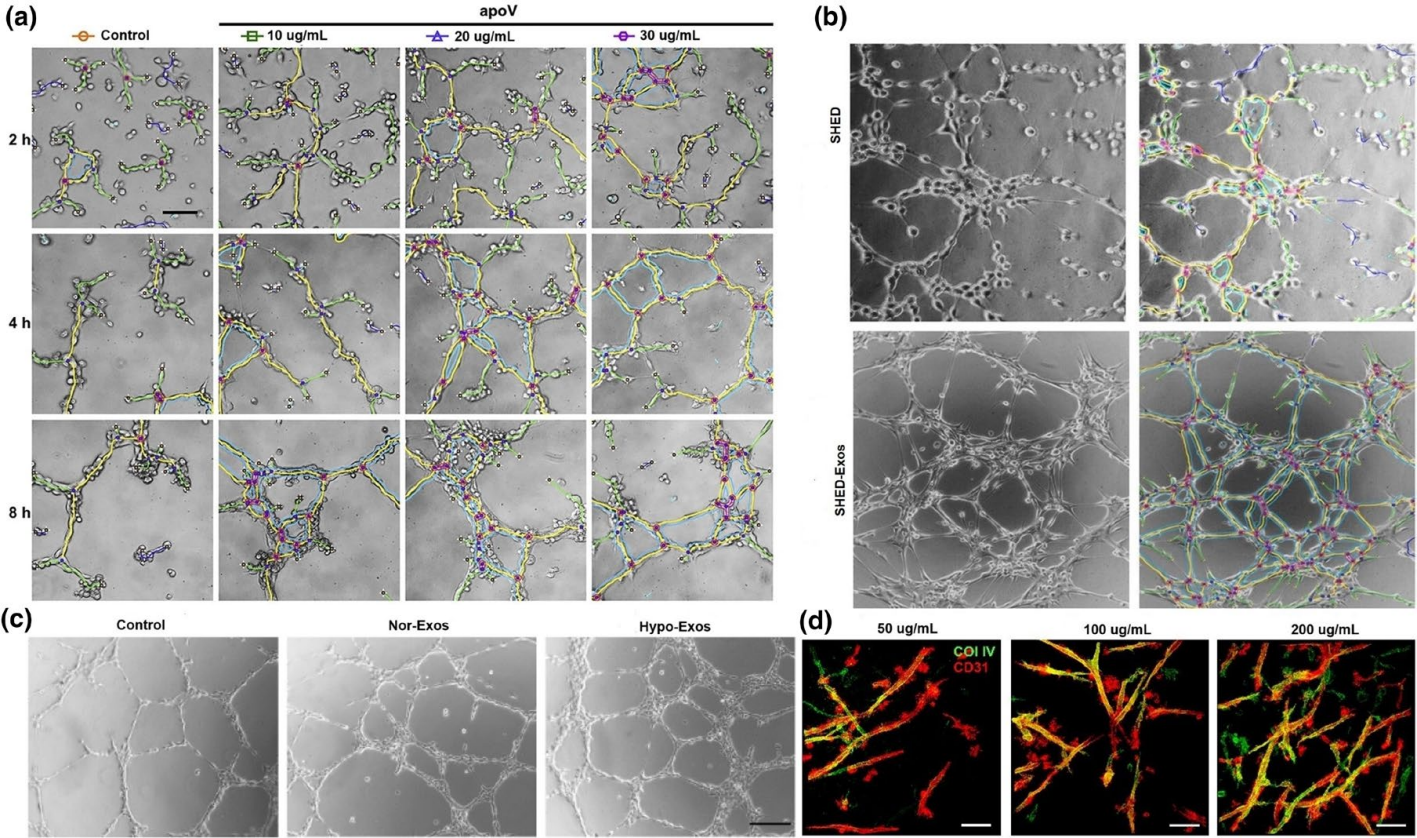
(a) Matrigel analysis demonstrating the effects of DPSC‐Exos (at concentrations of 10, 20 and 30 μg/mL at 2‐, 4‐ and 8‐h timepoints) on the tube formation of endothelial cells (reprinted with permission from Li, Wu, et al., 2022); (b) Tube formation analysis showing the endothelial differentiation of SHED‐ and SHED‐Exos (reprinted with permission from Wu et al., 2021); (c) Tube formation effects of Nor‐Exos and Hypo‐Exos on HUVECs (reprinted with permission from Li, Xian, et al., 2022); (d) Dose‐dependent tube formation effects of DPSCs in fibrin gels containing increasing concentrations of exosomes, with the most extensive tubular network forming at an exosomal concentration of 200 μg/mL (reprinted with permission from Zhang, Thiebes, et al., 2020).

Similarly, SHED‐Exos demonstrated angiogenic capabilities by delivering mitochondrial Tu translation elongation factor and activating endothelial cells through the transcription factor EB‐autophagy pathway, culminating in the development of pulp‐like tissue abundant in vascular structures within a beagle model (Figure 2a) (Li, Wu, et al., 2022). Additional studies revealed that exosomes from hypoxia‐preconditioned DPSCs improved HUVEC angiogenesis through the upregulation of lysyl oxidase‐like 2 (LOXL2), which significantly improved tube formation and modified proteomics profiles (Figure 2c) (Li, Xian, et al., 2022). Moreover, studies have indicated that exosomes derived from periodontitis‐affected DPSCs enriched with miR‐378a increase endothelial cell angiogenesis by downregulating the expression of suppressor of fused homologue (Sufu) and activating the Hedgehog/glioma‐associated oncogene 1 (Gli1) signalling pathway (Zhou et al., 2021). These results underscore the capacity of DSC‐Exos to convey bioactive molecules that directly modulate pathways associated with angiogenesis.

**FIGURE 3:**
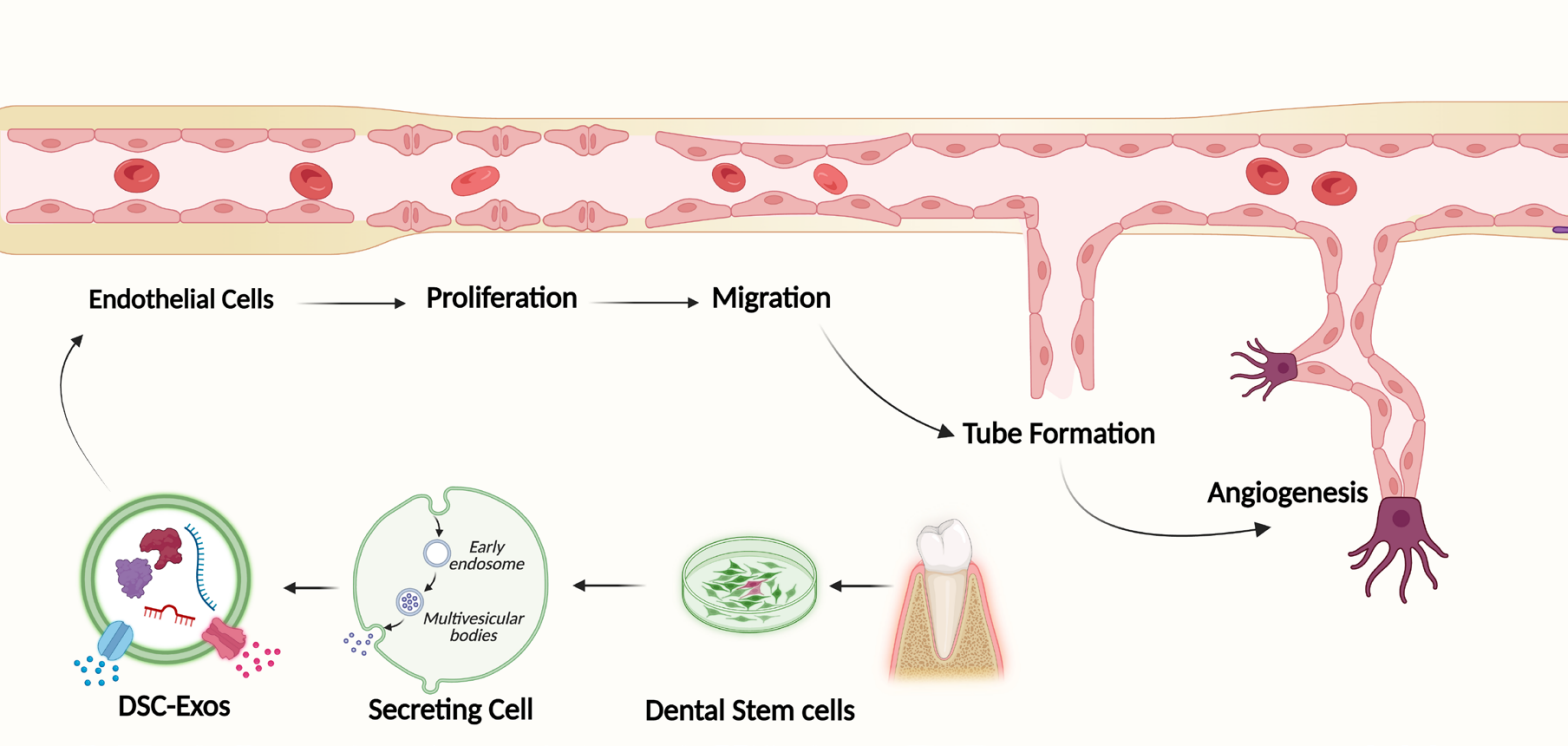
Mechanism of angiogenesis mediated by the DSC‐Exos. DSCs secrete exosomes that promote endothelial cell proliferation, migration and tube formation, ultimately contributing to angiogenesis (reprinted with permission from Zou et al., 2023).

A novel injectable exosome‐fibrin gel delivery platform demonstrated effective and sustained release of DPSC‐Exos, promoting the rapid formation of vascular‐like structures and collagen deposition in vitro, thus presenting a minimally invasive approach for regenerative endodontics (Figure 2d) (Zhang, Thiebes, et al., 2020). Exosomes derived from DPSCs under conditions that promote angiogenic differentiation have profound effects on cellular proliferation, migration and the expression of angiogenic markers, which are facilitated by pivotal miRNAs that increase cell homing and differentiation (Ganesh et al., 2022). Similarly, SHED aggregate‐Exos enriched with miR‐26a promoted angiogenesis by modulating TGF‐β/SMAD2/3 signalling, thereby facilitating successful regenerative endodontics (Figure 2b) (Wu et al., 2021). Moreover, exosomes derived from dental pulp cells promoted angiogenesis in HUVECs by increasing proliferation, proangiogenic factor expression and tube formation, with p38 MAPK signalling inhibition further augmenting tubular morphogenesis (Xian et al., 2018). The findings of this review highlight the pivotal importance of DSC‐Exo‐based therapies in facilitating angiogenesis and pulp regeneration by modulating endothelial cell function and promoting vascularization (Table 3). Figure 3 depicts the mechanism of angiogenesis mediated by DSC‐Exos.

**TABLE 3:** Summary of studies assessing the role of exosomes derived from dental stem cells in angiogenesis.

Study	Objective	Exosome/miRNA source	Pathway	Application	Key findings
Liu et al. (2023)	To investigate the angiogenic effects of hypoxic SCAP‐Exos on HUVECs for dental pulp regeneration	Exos from hypoxia‐preconditioned SCAPs	HIF‐1α/JAG1/VEGF signalling pathway activated; JAG1 delivered via Exos increased VEGF production	Promotes angiogenesis, offering potential for vascular reconstruction in dental regeneration	Hypoxic SCAP‐Exos enhanced HUVEC proliferation, migration and tube formation
Li, Duan, et al. (2022)	To explore the role of Exos from human deciduous pulp stem cells in dental pulp regeneration	Exos from human deciduous pulp stem cells	Exos‐carried mitochondrial Tu translation elongation factor regulated angiogenesis via TFEB‐autophagy pathway	Promoted dental pulp‐like tissue formation and revascularization in a beagle model	Exos enhanced endothelial cell angiogenesis and facilitated pulp revascularization
Li, Xian, et al. (2022)	To assess the angiogenic potential of hypoxia‐preconditioned DPSC‐Exos	Exos from normoxic and hypoxic DPSCs	LOXL2‐mediated angiogenesis through hypoxia‐induced proteome changes	Demonstrated enhanced angiogenesis and potential application in dental pulp regeneration	Hypo‐Exos enhanced HUVEC proliferation, migration and angiogenesis; LOXL2 identified as a key protein
Zhang, Thiebes, et al. (2020)	To develop an injectable Exos‐fibrin gel system for promoting angiogenesis in dental pulp regeneration	DPSC‐Exos integrated into a fibrin gel	Exos enhanced VEGF release, leading to rapid angiogenesis	Injectable gel system offers minimally invasive strategy for regenerative endodontic therapy	Exos‐fibrin gels promoted vascular‐like structure formation and collagen deposition
Ganesh et al. (2022)	To determine the effect of DPSC‐Exos on cell homing and angiogenic differentiation for pulp regeneration	DPSC‐Exos under growth or angiogenic conditions	Key exosomal miRNAs and angiogenic markers (VEGFA, FLT1, PECAM1) identified	Significant therapeutic potential for exosome‐based cell homing and angiogenic differentiation	DPSC‐Exos enhanced proliferation, migration and angiogenic marker expression
Xian et al. (2018)	To investigate the role of DPC‐Exos in angiogenesis	Exosomes from dental pulp cells	p38 MAPK signalling inhibition enhanced DPC‐Exos‐induced angiogenesis	Highlighted the role of DPC‐Exos in angiogenesis with potential applications in regenerative medicine	DPC‐Exos promoted HUVEC proliferation, proangiogenic factor expression and tube formation

Study	miRNA involved	Key genes involved	Regulatory mechanism	Significance	Key findings
Zhou et al. (2021)	miR‐378a	Sufu, Gli1, VEGF	miR‐378a downregulates Sufu, activating the Hedgehog/Gli1 signalling pathway and enhancing angiogenesis‐related factor expression	Highlights the role of Exos‐derived miR‐378a in promoting cell angiogenesis, offering a new target for enhancing vessel regeneration through modified stem cells or Exos	P‐Exos carrying miR‐378a promote endothelial cell proliferation, migration and tube formation
Wu et al. (2021)	miR‐26a	TGF‐β, SMAD2/3	miR‐26a enriched in SA‐Exos regulates TGF‐β/SMAD2/3 signalling to improve angiogenesis in SHED and HUVECs	Offers insights into the role of SA‐Exos and miR‐26a in pulp regeneration, supporting the development of new regenerative strategies	SA‐Exos significantly improved pulp tissue regeneration and angiogenesis in vivo

### Inflammation and immune regulation

Inflammation has historically been underestimated in the context of pulp healing and regeneration, previously regarded solely as an adverse consequence. Current research indicates that pulp inflammation is essential for regenerative endodontics (Goldberg et al., 2015). Rather than being a detrimental process, controlled inflammation serves as a prerequisite for initiating repair and coordinating the actions of immune cells, MSCs and angiogenic factors (Han et al., 2022; Karin & Clevers, 2016). Initial investigations attributed the anti‐inflammatory and immunosuppressive properties of MSCs to their direct interactions with immune cells (Laranjeira et al., 2015). Nonetheless, MSCs exhibit a lifespan of less than a week following systemic administration, whilst their therapeutic benefits endure, implying that the immune functionality of MSCs is at least partially attributable to their paracrine effects, with exosomes likely serving as their primary active agents (Harrell et al., 2019; Zhang et al., 2014). It was concluded that DSC‐Exos demonstrate anti‐inflammatory and immunomodulatory effects primarily through four mechanisms (Zou et al., 2023): (i) diminishing inflammatory responses; (ii) modulating the macrophage phenotype and activity; (iii) influencing T‐cell and B‐cell behaviour; and (iv) altering the dendritic cell phenotype and promoting natural killer cell proliferation.

On the basis of the findings of this review, the application of DSC‐Exos and exosomal miRNAs has considerable potential in regenerative endodontics through the modulation of inflammatory responses and the facilitation of tissue repair. MSC‐Exos, such as those derived from DPSCs, DFSCs, SCAPs and umbilical cord mesenchymal stem cells (UCMSCs), have substantial anti‐inflammatory and reparative effects within the inflamed dental pulp environment. DFSC‐Exos exhibited antioxidative and pro‐healing properties, effectively alleviating oxidative stress within dental pulp tissues (Li et al., 2024). In a model of pulpitis characterized by inflammation, DFSC‐Exos promoted cell survival, proliferation and odontogenic processes whilst restoring mitochondrial equilibrium. When incorporated into a hydrogel scaffold designed for controlled release, DFSC‐Exos facilitated synergistic antioxidant actions and increased pulp tissue repair (Li et al., 2024). Compared with DPSC‐Exos, UCMSC‐Exos demonstrated superior efficacy in mitigating inflammation, reversing apoptotic processes and modulating cytokine expression under LPS‐induced inflammatory conditions (Zeng et al., 2022). SCAP‐Exos improved pulp inflammation by increasing the conversion of regulatory T cells (Tregs) through Tet methylcytosine dioxygenase 2 (Tet2)‐mediated demethylation of forkhead box P3 (Foxp3), which stabilized Treg expression and modified the immune microenvironment to be conducive to regenerative endodontics (Yu et al., 2022). In mild pulp inflammation, LPS‐preconditioned DPSC‐Exos significantly increased tissue regeneration, promoting functional healing that approximated normal pulp architecture (Chen et al., 2021).

**FIGURE 4:**
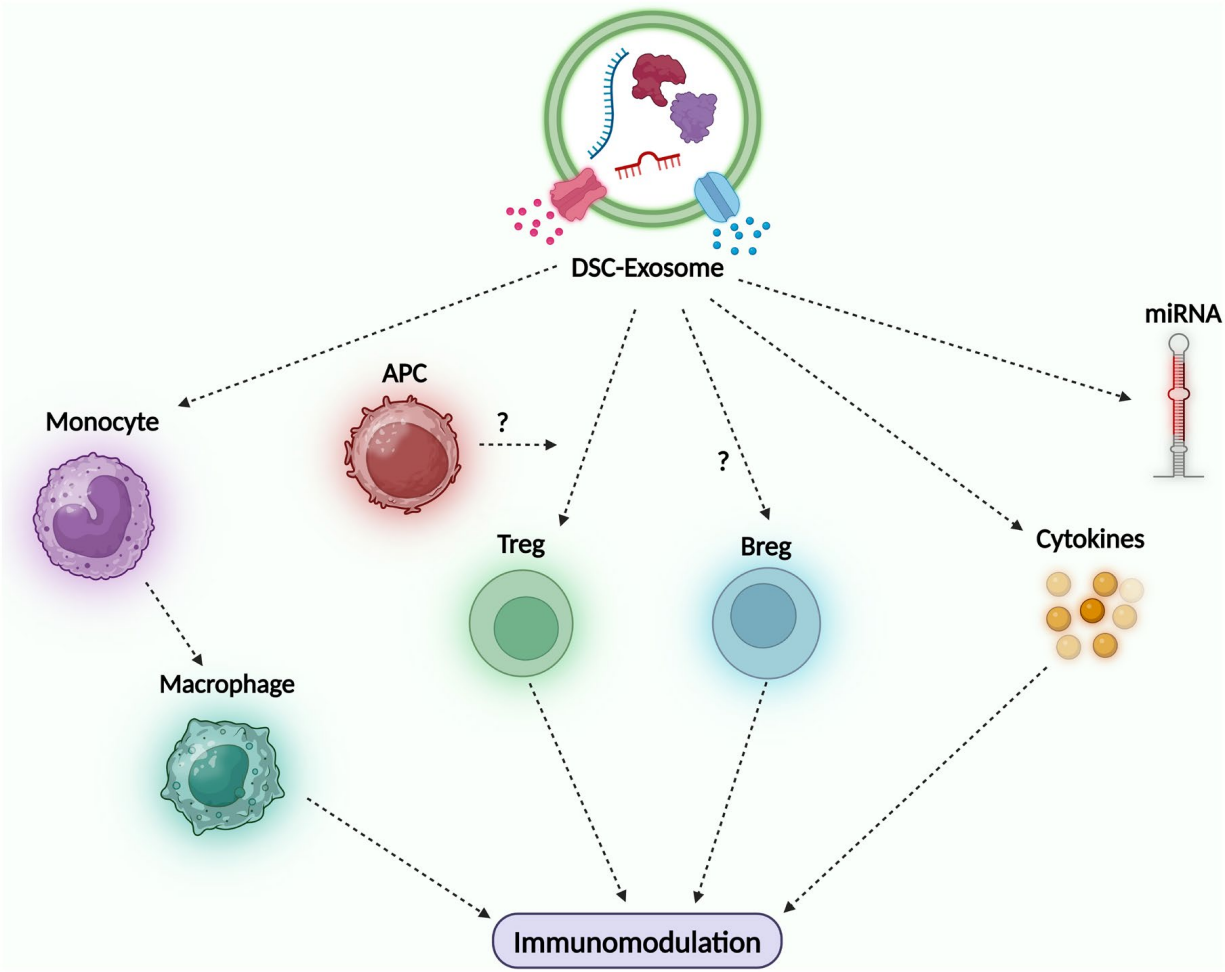
Immunomodulatory effects of the DSC‐Exos. DSC‐Exos influence immune cell populations, including monocytes, macrophages, antigen‐presenting cells (APCs), regulatory T cells (Tregs) and regulatory B cells (Bregs). Through the secretion of miRNAs and cytokines, DSC‐Exos modulate immune responses, contributing to immunoregulation. The exact mechanisms of interaction with certain cell types (i.e., Bregs) remain to be elucidated.

miR‐125a‐3p, which is significantly upregulated in DPSC‐Exos, facilitates the transition of macrophages to the pro‐healing M2 phenotype by inhibiting the nuclear factor kappa B (NF‐κB) and TLR signalling pathways (Wang, Zheng, et al., 2020). This miRNA also promotes odontogenesis through the release of bone morphogenetic protein 2 (BMP2) from macrophages (Zheng et al., 2020). Similarly, let‐7c‐5p inhibits the DMP1‐mediated NF‐κB pathway, thereby diminishing LPS‐induced inflammatory responses and safeguarding pulp tissues (Yuan et al., 2018). Another study revealed that let‐7c‐5p promoted anti‐inflammatory and pro‐osteogenic effects in DPSCs by restoring the proliferation and osteogenic differentiation impaired by pulpitis through the inhibition of high mobility group A2 (HMGA2)/phosphatidylinositol 3‐kinase (PI3K)/Akt signalling (Yuan et al., 2019). Circular RNAs (circRNAs), such as circ_0138960, exacerbate inflammation by sequestering miR‐545‐5p, which modulates myeloid differentiation primary response 88 (MYD88) expression and NF‐κB activation (Liang et al., 2023). The knockdown of circ_0138960 results in a reduction in inflammation and oxidative stress within dental pulp cells (Liang et al., 2023). Moreover, miR‐181a was shown to regulate interleukin (IL‐8), a proinflammatory cytokine whose expression is elevated during inflammatory conditions, through direct binding to its 3′UTR (Galicia et al., 2014). This interaction effectively decreases cytokine expression, underscoring the potential of miR‐181a to influence the inflammatory response within dental pulp fibroblasts (Galicia et al., 2014) (Table 4). Thus, a reciprocal relationship exists between inflammation and differentiation, where immune‐modulating exosomes not only mitigate inflammatory damage but also prime MSCs for subsequent odontogenic and angiogenic activities. Figure 4 depicts the mechanism of immunomodulation mediated by the DSC‐Exos.

**TABLE 4:** Summary of studies assessing the role of exosomes derived from dental stem cells in inflammation and immune response.

Study	Objective	Exosome/miRNA source	Pathway	Application	Key findings
Li et al. (2024)	To explore the antioxidative and pro‐healing effects of DFSC‐Exos on a rat pulpitis model	Dental follicle stem cell‐derived Exos	Antioxidative effects and mitochondrial protection using SA‐RhB hydrogel	DFSC‐Exos‐loaded hydrogel provides a minimally invasive treatment for pulpitis	DFSC‐Exos enhanced survival, proliferation and odontogenesis of H_2_O_2_‐injured DPSCs and restored mitochondrial oxidative balance DFSC‐Exos‐loaded SA‐RhB hydrogel provided controlled release and synergistic antioxidant effects
Zeng et al. (2022)	To evaluate the effects of UCMSCs‐Exos and DPSCs‐Exos on LPS‐induced inflammation in DPSCs	UCMSCs‐Exos and DPSCs‐Exos	Regulation of proinflammatory and anti‐inflammatory cytokine balance	Alleviates inflammation in DPSCs and highlights the potential of UCMSCs‐Exos in clinical applications	Both Exos reversed LPS‐induced effects on proliferation, apoptosis and cytokine secretion, with UCMSCs‐Exos showing stronger effects
Chen et al. (2021)	To evaluate the effects of Exos from LPS‐preconditioned DPSCs on dental pulp regeneration	Exos from LPS‐preconditioned human dental pulp stem cells	Paracrine modulation of regenerative responses in a mild inflammatory environment	Potential applications in regenerative endodontics through functional pulp regeneration	L‐Exos enhanced BMSC proliferation, migration, angiogenesis, differentiation and facilitated functional pulp regeneration with better outcomes than N‐Exos
Yu et al. (2022)	To evaluate the effects of SCAP‐Exos on experimentally induced pulpitis and their role in Treg conversion	Exosomes derived from stem cells of the apical papilla	Tet2‐mediated Foxp3 demethylation stabilizes Foxp3 expression	SCAP‐Exos regulate immune microenvironments to promote regeneration and offer a cell‐free approach for early pulp inflammation treatment	SCAP‐Exos alleviated inflammation, promoted Treg conversion and enhanced Tet2‐mediated Foxp3 demethylation

Study	miRNA involved	Key genes involved	Regulatory mechanism	Significance	Key findings
Yuan et al. (2019)	let‐7c‐5p	HMGA2, PI3K, Akt, RUNX2, OCN, OPN, OSX, MSX2	Let‐7c‐5p suppresses HMGA2/PI3K/Akt signalling, restoring osteogenic differentiation and cell proliferation	Demonstrates the potential of let‐7c‐5p as a therapeutic agent for pulpitis	Let‐7c‐5p improves osteogenesis and reduces inflammation in DPSCs during pulpitis by inhibiting HMGA2/PI3K/Akt pathway activation
Wang, Zheng, et al. (2020)	miR‐125a‐3p	Fyn, Neuropilin‐1	MiR‐125a‐3p downregulates Fyn, impacting NF‐κB signalling and inflammation modulation	Suggests miR‐125a‐3p as a therapeutic target for inflammation and dental caries	MiR‐125a‐3p inhibits Fyn, reducing inflammatory responses and promoting odontoblastic differentiation in DPSCs
Zheng et al. (2020)	miR‐125a‐3p	IKBKB, BMP2, TLR, NF‐κB	MiR‐125a‐3p targets IKBKB, enhancing BMP2 release in macrophages and promoting odontogenesis	Highlights DPSCs‐sEV and miR‐125a‐3p as biomimetic tools for odontogenesis	DPSCs‐sEV promote odontogenesis by shifting macrophages to the M2 phenotype via miR‐125a‐3p, which inhibits NF‐κB and TLR signalling
Liang et al. (2023)	miR‐545‐5p	MYD88, NF‐κB	Circ_0138960 sponges miR‐545‐5p to regulate MYD88, activating NF‐κB signalling	Provides insights into targeting circRNAs for pulpitis treatment	Circ_0138960 knockdown reduces LPS‐induced inflammation in hDPCs by regulating the miR‐545‐5p/MYD88/NF‐κB axis
Yuan et al. (2018)	let‐7c‐5p	DMP1, NF‐κB	Let‐7c‐5p downregulates DMP1, suppressing NF‐κB signalling and inflammation	Reinforces let‐7c‐5p's role in protecting dental pulp during inflammation	Let‐7c‐5p attenuates LPS‐induced pulpitis by inhibiting DMP1‐mediated NF‐κB pathway activation
Galicia et al. (2014)	miR‐181a	IL‐8	miR‐181a directly binds IL‐8 3′UTR, inversely modulating its levels during inflammation	Demonstrates miR‐181a's critical role in cytokine regulation and inflammation	MiR‐181a regulates IL‐8 expression by binding to its 3′UTR, controlling inflammatory responses in dental pulp fibroblasts

## LIMITATIONS AND FUTURE DIRECTIONS

The majority of the included studies used differential ultracentrifugation (Abdelgawad et al., 2022; Chen et al., 2021, 2022; Ivica et al., 2020; Li et al., 2024; Li & Ge, 2022; Li, Ju, et al., 2021; Li, Wu, et al., 2022; Li, Xian, et al., 2022; Liu et al., 2023; Lu et al., 2024; Luo et al., 2023; Swanson et al., 2020; Wang et al., 2023; Wang, Yang, et al., 2019; Wu et al., 2021; Xian et al., 2018; Yang et al., 2022; Yu et al., 2022; Zeng et al., 2022; Zhang, Thiebes, et al., 2020; Zhou et al., 2021; Zhuang et al., 2020), whilst a few of them utilized polymer‐based precipitation (Diomede et al., 2022; Ganesh et al., 2022; Hu et al., 2019; Huang et al., 2016) and ultrafiltration (Zhang, Yang, et al., 2020) methods to isolate DSC‐Exos. Current limitations include the lack of a standardized protocol for the isolation of DSC‐Exos to facilitate odontoblastic differentiation in a clinical environment and an inadequate comprehension of the exact molecular mechanisms that mediate the relationship between these DSC‐Exos and odontoblasts. Moreover, the inconsistencies in exosome effectiveness attributable to variations in donor cell types, preconditioning strategies and isolation methodologies present a significant obstacle, whilst the scarcity of in vivo investigations limits their applicability from pre‐clinical results to clinical settings. To mitigate these challenges, initiatives should concentrate on establishing standardized and reproducible methodologies for the isolation and preconditioning of DSC‐Exos. Comprehensive multiomics investigations are imperative to clarify the signalling pathways and molecular interactions that are relevant to odontogenic differentiation. Extensive in vivo research and clinical trials are necessary to substantiate therapeutic efficacy, alongside the examination of gene‐editing technologies, including clustered regularly interspaced short palindromic repeats and CRISPR‐associated protein 9 (CRISPR)/Cas9 technology, to improve the regenerative capacities of exosomes by influencing critical pathways such as the TGF‐β1/SMAD and Wnt/β‐catenin pathways. Additionally, the incorporation of sophisticated delivery mechanisms, including biomimetic scaffolds and hydrogels, can facilitate the targeted and sustained release of these exosomes at sites of injury.

Research limitations are evident in the inadequacy of investigations focused on the function of DSC‐Exos in neurogenesis within the pulp–dentine complex and in the limited analysis of the intercommunication between odontogenic and neurogenic signalling pathways facilitated by exosomes. Moreover, the bioactive components within exosomes that play vital roles in neuroprotection and neuroregeneration are poorly understood. To overcome these limitations, it is imperative to broaden research endeavours concerning exosomal cargo, particularly specific miRNAs, lncRNAs and proteins that affect neurogenic mechanisms. Further examination of the collaborative effects between odontogenic and neurogenic signalling pathways is essential to improve the functional rehabilitation of the pulp–dentine complex. Advanced in vitro coculture models and organ‐on‐chip platforms should be used to investigate the dynamics between exosomes and neural cells. Bioengineered exosomes or nanovesicles with optimized cargo might be synthesized for precise neuroregenerative interventions.

The clinical applicability of DSC‐Exo‐centric regenerative endodontic therapies presents a formidable challenge, which is compounded by a deficiency in understanding the mechanisms through which these exosomes influence angiogenesis in compromised pulp niches. Additionally, robust delivery mechanisms to facilitate vascular integration within the restricted pulp cavity are urgently needed. These obstacles might be overcome through the implementation of hypoxic preconditioning and genetic modifications aimed at amplifying the angiogenic capabilities of the exosomes. An exploration of particular angiogenic miRNAs, such as miR‐26a and miR‐378a, could refine therapeutic strategies. The combination of injectable hydrogels and nanocomposite scaffolds to ensure sustained exosome release and increased vascularization represents another promising approach. The advancement of high‐throughput biomanufacturing techniques is essential for enabling the scalable production of DSC‐Exos that maintain consistent quality and efficacy.

Current knowledge regarding the long‐term implications of DSC‐Exo‐based regenerative endodontic therapies for immune modulation is limited, with few investigations focusing on the interaction between inflammatory and regenerative mechanisms within pulp tissues. Moreover, the variability inherent in the immunomodulatory characteristics of exosomes, contingent upon the source of the cells and the inflammatory microenvironment, adds further complexity. To address these limitations, the exploration of novel exosomal miRNAs, such as let‐7c‐5p and miR‐125a‐3p, is warranted because of their potential roles in immune regulation and tissue repair. The integration of DSC‐Exo therapy with anti‐inflammatory agents may provide an effective means to modulate inflammatory responses. Longitudinal studies are necessary to assess the safety and efficacy of these therapeutic interventions and personalized strategies that tailor exosomal content to specific inflammatory profiles could enhance therapeutic outcomes.

The evidence elucidating the mechanisms by which DSC‐Exos mitigate apoptosis in odontoblasts and adjacent pulp cells under chronic inflammatory conditions remains sparse, and the ramifications of senescence‐associated secretory phenotypes in regenerative endodontics are inadequately understood. In‐depth mechanistic investigations are needed to elucidate how exosomal cargo influences apoptosis and autophagy pathways in odontoblasts. The development of senolytic agents (i.e., drugs that target and eliminate senescent cells, which are cells that accumulate with age) or engineered DSC‐Exos aimed at decreasing the detrimental effects of cellular senescence could bolster therapeutic efficacy. Combinatorial strategies, such as the integration of exosomal therapy with bioactive molecules that increase cellular resilience to oxidative stress and senescence, show considerable promise.

Our understanding of the mechanisms through which exosomes facilitate the migration and proliferation of DSCs to the injured pulp–dentine interface remains incomplete, with limited research on the modulation of signalling pathways such as the Notch and Akt pathways. Bridging these knowledge gaps necessitates the investigation of specific miRNAs, such as miR‐140‐5p and miR‐584, that govern the DSC behaviours essential for regenerative endodontics. The enhancement of DSC‐Exo‐mediated signalling pathways via the use of small molecules or genetic tools with the aim of optimizing the recruitment and proliferation of these cells is needed. The incorporation of advanced imaging methodologies is also essential for tracking DSC migration and proliferation in vivo during exosome‐based therapies. Furthermore, the development of DSC‐Exo‐integrated bioactive materials, including fibrin gels, could significantly increase DSC recruitment and enhance their regenerative potential.

By systematically addressing these constraints, the therapeutic potential of DSC‐Exo‐based regenerative endodontic therapies can be fully realized, facilitating comprehensive structural and functional restoration of the pulp–dentine complex.

## AUTOLOGOUS VERSUS ALLOGENEIC EXOSOMES IN REGENERATIVE ENDODONTICS

### Autologous exosomes

A pivotal question regarding the potential clinical utilization of exosomes in regenerative endodontics pertains to the preference for exosome‐based therapies to be autologous (i.e., originating from the patient's own DSCs) (Zhao et al., 2021) or allogeneic (i.e., sourced from external donors) (Gonzalez‐Nolasco et al., 2018). Although both modalities exhibit considerable promise, they entail unique advantages and challenges that necessitate meticulous evaluation to facilitate optimal therapeutic outcomes.

Autologous exosomes confer several significant advantages. Given that autologous exosomes are derived from an individual's own cellular material, the likelihood of immune rejection or adverse immune reactions is substantially reduced (Kong, Wang, et al., 2024). This phenomenon is particularly crucial in regenerative endodontics, where the introduction of foreign exosomes could incite inflammation, thereby jeopardizing pulp regeneration (Hammouda et al., 2023). Autologous exosomes can be specifically tailored to address the distinct regenerative requirements of the patient's dental tissue (Xing et al., 2020), thereby ensuring a more precise and efficacious therapeutic strategy. The exosomal profile from the patient may harbour unique signalling molecules that are optimally aligned with their pulp microenvironment (Chen et al., 2022). The use of autologous exosomes mitigates the potential risk associated with the transmission of infections or diseases from donor tissues (Van Delen et al., 2024), which is a significant concern in allogeneic applications. The regulatory approval pathways for autologous therapies may be more streamlined than those of their allogeneic counterparts, as the associated risks of complications linked to foreign biological materials are diminished. Ethical concerns related to donor cell procurement are also significantly reduced (Hamad et al., 2024).

Despite these benefits, the application of autologous exosomes is accompanied by several limitations that may impede their broader clinical utility. The process of preparing autologous exosomes necessitates patient‐specific stem cell isolation, culture expansion and exosome extraction, which can be both costly and time‐consuming (Kong, Wang, et al., 2024). In contrast, allogeneic exosomes can be produced in advance and stored for prompt application. The regenerative capacity of exosomes may differ amongst patients due to their age, health status and genetic predisposition (Xing et al., 2020). For example, stem cells harvested from younger patients may yield exosomes with greater regenerative potential than those obtained from older individuals. Moreover, not every patient may possess a viable source of DSCs, particularly in instances of severe pulp necrosis or advanced dental pathology (Ivica et al., 2021). In such scenarios, allogeneic exosomes may serve as a viable alternative source.

### Allogeneic exosomes

In light of the restrictions associated with autologous therapy, allogeneic exosomes may offer a more scalable and clinically practical solution. They can be produced in bulk, preserved and made readily accessible for clinical application (Ivica et al., 2021), thus providing an immediate therapeutic option for patients requiring regenerative endodontics. The large‐scale production of allogeneic exosomes facilitates increased quality control, ensuring uniform therapeutic potency and biomolecular composition (Kong, Wang, et al., 2024). This approach diminishes the variability encountered in autologous preparations. They can be derived from highly effective stem cell populations, such as young donors or preconditioned stem cells, to optimize their regenerative performance. Furthermore, the mass production of allogeneic exosomes has the potential to decrease the overall expenses associated with regenerative therapies in contrast to the individualized production necessitated by autologous methods (Kong, Wang, et al., 2024).

A significant concern surrounding allogeneic exosomes is their probability of immune recognition and subsequent clearance (Prunevieille et al., 2021). Nevertheless, several investigations have indicated that exosomes possess low immunogenic properties because of the absence of major histocompatibility complex (MHC) molecules (Exosomes, 2004; Hiltbrunner et al., 2016; Vincent‐Schneider et al., 2002). There are essential strategies to improve the biocompatibility of allogeneic exosomes: (1) altering exosomal membranes to diminish immune activation and extend the duration of circulation; (2) preconditioning the recipient's immune system to accept allogeneic exosomes without instigating inflammatory responses; and (3) assessing donor‐derived exosomes to reduce the risk of immunological incompatibilities.

### Future perspectives

Considering the unique benefits and obstacles presented by both autologous and allogeneic exosome‐based therapies, the following research endeavours should be emphasized: (1) executing clinical trials to directly evaluate the efficacy, safety and immunogenicity of autologous therapy against allogeneic exosome therapy within regenerative endodontics; (2) formulating standardized methods for exosome extraction, characterization and preservation to ensure uniform therapeutic outcomes; (3) examining hybrid methodologies, such as integrating autologous and allogeneic exosomes or utilizing patient‐derived cells preconditioned with allogeneic exosomes to bolster regenerative potential; and (4) instituting explicit guidelines for the clinical application of exosome‐based therapies to ensure safety, efficacy and adherence to ethical standards.

## A NOVEL COMPREHENSIVE MODEL FOR REGENERATIVE ENDODONTICS

**FIGURE 5:**
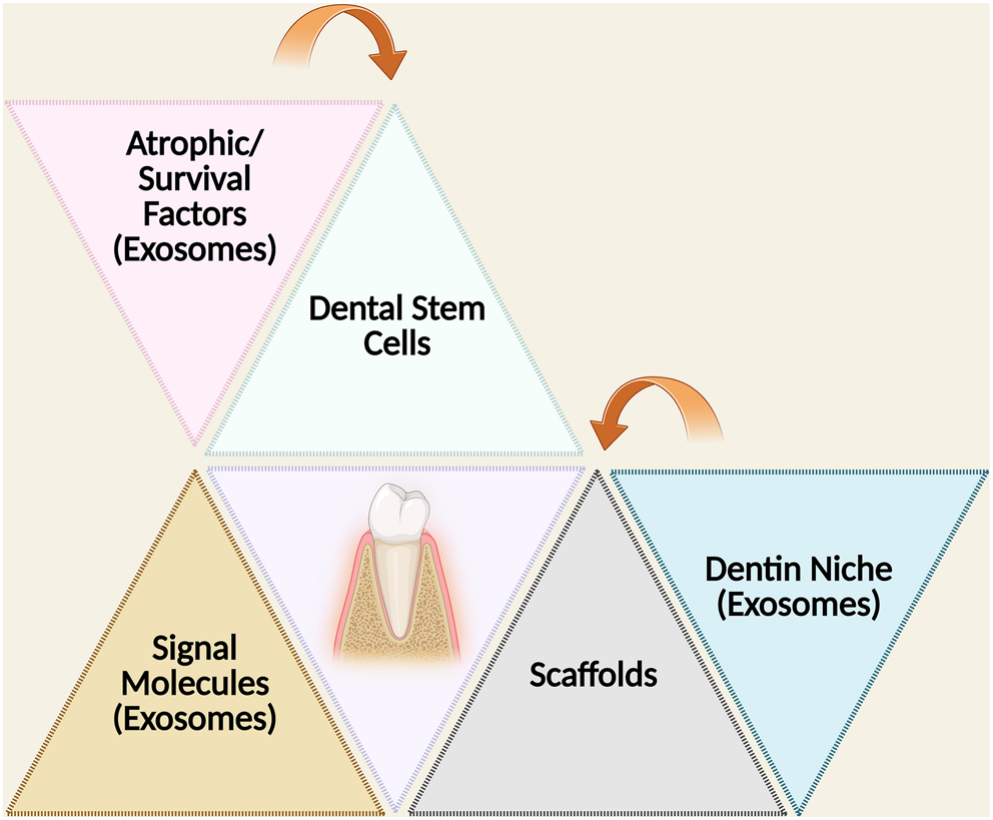
A schematic illustrating an innovative approach to regenerative endodontic therapies, integrating three traditional (i.e., dental stem cells, signal molecules including exosomes and scaffolds) and two contemporary elements (i.e., dentine niche including exosomes and atrophic/survival factors including exosomes). The arrows represent the interactions and influences exerted on DSC within their reconstructive niches.

Based on the findings of this review, Figure 5 depicts a conceptual framework of an innovative regenerative endodontic therapy approach, amalgamating both traditional and contemporary components to reconstruct a viable dental microenvironment with an emphasis on holistic regenerative endodontics that has been presented. The elements are arranged in a triangular framework, demonstrating their interdependence and influence on DSCs and odontoblastic differentiation.

### Traditional triad

#### Dental stem cells

These cells serve as the principal agents of regeneration and possess the capacity for multilineage differentiation, which is fundamental for the repair of dental tissues. Their activation and subsequent differentiation into odontoblasts are crucial for the processes of dentinogenesis and the regeneration of pulp tissue. This process includes the formation of tubular dentine, thereby reinstating the structural and functional integrity of the pulp–dentine complex.

#### Signalling molecules

This category includes cytokines and growth factors, such as BMP2, TGF‐β1 and various exosome‐mediated miRNAs (including miR‐223‐3p and miR‐148a‐3p), that modulate odontoblastic differentiation, mineralization and cellular activities, including proliferation and migration. Signalling molecules also direct lineage‐specific differentiation, which is essential for the establishment of a protective dentinal barrier.

#### Scaffolds

Scaffolds, such as decellularized pulp matrix, thermoresponsive hydrogels and photocrosslinkable biomaterials, act as physical support to bolster cell attachment, proliferation and differentiation. Furthermore, these scaffolds facilitate the sustained delivery of therapeutic agents, including exosomes, thereby enhancing targeted regenerative efforts. Importantly, scaffolds also serve as carriers of ECM constituents and biochemical cues, actively contributing to the formation of a bioactive dentine niche that modulates cellular responses.

### Contemporary triad

#### Dentin niche

The dentine niche represents a specialized microenvironment that includes ECM constituents, odontoblasts and biochemical signals, all of which influence the behaviour and differentiation of DSCs. The interaction between scaffolds and ECM components within this niche plays a pivotal role in creating a bioactive matrix that facilitates odontogenesis and tissue regeneration. Research has indicated that scaffolds enriched with exosomes within the dentine niche can considerably increase odontogenesis and tissue regeneration through signalling pathways such as the p38 MAPK and Wnt/β‐catenin pathways.

#### Trophic/survival factors

This category includes exosomes and engineered nanoparticles that increase the survival and functionality of DSCs. For example, SCAP‐Exos effectively promote odontoblastic differentiation and the formation of dentin bridges by delivering miRNAs and activating signalling cascades such as TGF‐β1/SMAD and ERK. Scaffolds embedded with trophic factors and exosomes contribute to maintaining the viability of DSCs within the dentine niche, hence reinforcing their regenerative potential.

#### Exosomes

Exosomes generated under odontogenic conditions significantly promote odontoblastic differentiation and mineralization. For example, exosomal miR‐27a‐5p diminishes the activity of inhibitory factors, whereas miR‐21 increases the expression of regenerative markers under inflammatory conditions. LncRNAs, such as CALB2 and IGFBP7‐AS1, regulate miRNA functionality to further facilitate regeneration. Furthermore, scaffold‐mediated exosome delivery ensures their localized and sustained release within the dentine niche, optimizing their regenerative potential and reinforcing the synergy amongst scaffolds, ECM components and biochemical signalling.

## CONCLUSION

This review highlights the immense potential of DSC‐Exos in facilitating the regeneration of the pulp–dentine complex. Through their bioactive components, exosomes significantly facilitate a diverse array of regenerative activities, including odontoblastic differentiation, dentine mineralization, neuroprotection, immune modulation, angiogenesis, cell proliferation, migration, apoptosis and senescence, all of which are vital for pulp healing and regeneration. In particular, the exosome‐mediated alteration of signalling pathways such as the p38 MAPK, TGF‐β1/SMAD and Wnt/β‐catenin pathways intensifies their regenerative efficacy. Moreover, DSC‐Exos exhibit marked anti‐inflammatory and immune‐modulatory characteristics, which are essential for addressing the inflammatory responses that frequently hinder successful regenerative endodontics. Their ability to recruit and activate endogenous MSCs, along with their influence on cellular activities such as proliferation, migration and apoptosis, positions exosome‐based therapies as promising modalities for enhancing clinical methodologies in regenerative endodontics. Importantly, the amalgamation of exosomes with innovative delivery systems, such as hydrogels, presents the potential for prolonged and targeted therapeutic results, thereby increasing both the effectiveness and biocompatibility of treatments. These results provide a compelling basis for future research into DSC‐Exo‐based therapies as viable, cell‐free alternatives to traditional regenerative approaches in endodontics.

## AUTHOR CONTRIBUTIONS

P.A. was responsible for choosing the topic, conducting the literature search, extracting and analysing the data, interpreting the results, updating the reference lists, writing the manuscript and reviewing the manuscript. N.E., N.F. and Y.Z. were responsible for designing the study, extracting and analysing the data, reviewing the data, interpreting the results and reviewing the manuscript. R.J.M. was responsible for supervision, review and editing. All the authors critically reviewed and approved the final draft and are responsible for the content and similarity index of the manuscript.

## FUNDING INFORMATION

The authors have nothing to report.

## CONFLICT OF INTEREST STATEMENT

All the authors declare that they have no conflicts of interest.

## ETHICS STATEMENT

Ethical approval was not needed, as this article does not contain any research involving human or animal experiments.

## Data Availability

All relevant data are presented in the manuscript.
